# P02.50. Naturopathy and yoga based life style intervention for cardiovascular risk reduction in patients with cardiovascular risk factors: a pilot study

**DOI:** 10.1186/1472-6882-12-S1-P106

**Published:** 2012-06-12

**Authors:** B Nandakumar, A Kadam, H Srikanth, R Rao

**Affiliations:** 1 Institute of Naturopathy and Yogic Sciences Medical Research Society, Bangalore, India

## Purpose

Lifestyle interventions have been known to reduce coronary risk in subjects with high risk for coronary heart disease (CHD). In this study we evaluated the effects of a residential yoga and naturopathy based lifestyle intervention on coronary risk reduction in subjects with high risk for CHD.

## Methods

In this randomized waitlisted controlled clinical trial, 72 subjects with known cardiovascular risk factors were randomized to receive residential 3 week yoga and naturopathy intervention (n=37) or waitlisted (n=35) to receive the same later. Outcome measures like resting blood pressure, Body Mass Index, lipid profile, fasting and post prandial blood glucose, and psychological measures such as Hospital Anxiety and Depression Scale (HADS), the Somatization component of SCL90, and General Health Perception Questionnaire were assessed at baseline and after the intervention waitlist period. Data were analysed between groups using ANCOVA with respective baseline measure as a covariate.

## Results

Compared to waitlist control, subjects in the Intervention group showed significantly (p < 0.05) lower adjusted mean values of systolic blood pressure (140.36 vs. 124.62), diastolic blood pressure (85.28 vs.76.93), fasting blood glucose (142.29 vs. 116.61), postprandial blood glucose (233.2 vs. 172.19), body mass index (33.05 vs. 31.86), total cholesterol(181.61 vs 161.04), LDL cholesterol(107.76 vs 85.72 ),triglycerides (152.8 vs. 131.74), anxiety (6.79 vs 4.98), depression (6.54 vs. 4.45) and somatization symptoms (7.84 vs. 3.56) at 3 weeks following intervention compared to waitlist controls. The intervention group showed significantly higher (p < 0.05) adjusted mean values of current health (29.56 vs 33.31) and prior health (9.67 vs 10.46) on the General Health Perception Questionnaire compared to waitlist controls following intervention.

## Conclusion

Naturopathy and Yoga based lifestyle interventions show promising results in reducing coronary risk indices in subjects at high risk for coronary heart disease. However, larger randomized controlled trials are needed to validate these findings.

